# Towards healthier and more sustainable diets in the Australian context: comparison of current diets with the Australian Dietary Guidelines and the EAT-Lancet Planetary Health Diet

**DOI:** 10.1186/s12889-022-14252-z

**Published:** 2022-10-19

**Authors:** Gilly A. Hendrie, Megan A. Rebuli, Genevieve James-Martin, Danielle L. Baird, Jessica R. Bogard, Anita S. Lawrence, Bradley Ridoutt

**Affiliations:** 1 CSIRO Health and Biosecurity, Adelaide, South Australia Australia; 2 CSIRO Agriculture and Food, St Lucia, QLD Australia; 3 University of Melbourne, Parkville, VIC Australia; 4 CSIRO Agriculture and Food, Clayton South, Victoria, Australia

**Keywords:** Food-based dietary guidelines, Dietary intakes, Diet quality, Sustainability, Environmental impacts

## Abstract

**Background:**

There is increasing focus on moving populations towards healthier and more environmentally sustainable dietary patterns. The Australian Dietary Guidelines provide dietary patterns that promote health and wellbeing. It is unclear how these guidelines align with the more recently published global recommendations of the EAT-Lancet Planetary Health Reference Diet, and how Australian diets compare to both sets of recommendations.

**Methods:**

Data from one 24-h recall collected for the 2011–13 National Nutrition and Physical Activity Survey were analysed for 5,920 adults aged 19–50 years. Subgroups of this population were identified by diet quality and lower or higher consumption of foods often considered to be environmentally intensive (higher animal meat and dairy foods) or associated with healthiness (higher vegetables and lower discretionary choices). Food group and nutrient composition of Australian diets were compared to diets modelled on the Australian Dietary Guidelines and Planetary Health Reference Diet. The environmental impacts of diets were estimated using an index of combined metrics.

**Results:**

Compared with the Planetary Health Reference Diet, the Australian Dietary Guidelines contained more servings of the vegetable, dairy and alternatives, fruit, and discretionary choices. The amount of meat and alternatives was higher in the Planetary Health Reference Diet than Australian Dietary Guidelines due to the inclusion of more plant-based meat alternatives. The average Australian diet contained two to almost four times the Australian Dietary Guidelines and Planetary Health Reference Diet maximum recommended intake of discretionary choices, and provided inadequate amounts of the vegetables, cereals, unsaturated fats and meats and alternatives food groups, primarily due to lower intakes of plant-based alternatives. The average Australian diet also contained less dairy and alternatives than the Australian Dietary Guidelines. In the average Australian diet, red meat and poultry contributed 73% to the total servings of meat and alternatives compared to 33% and 10% for the Australian Dietary Guidelines and Planetary Health Reference Diet respectively. The modelled Australian Dietary Guidelines diet met the relevant nutrient reference value for all 22 nutrients examined, whereas the Planetary Health Reference Diet contained an inadequate amount of calcium. The environmental impact scores of the Planetary Health Reference Diet and Australian Dietary Guidelines were 31% and 46% lower than the average Australian diet.

**Conclusions:**

Significant changes are required for Australians’ dietary intake to align more closely with national and global dietary recommendations for health and environmental sustainability.

**Supplementary Information:**

The online version contains supplementary material available at 10.1186/s12889-022-14252-z.

## Background

There has been a focus on moving towards a more sustainable food system, which has been described as one that delivers food security and nutritious foods for populations in a way that does not impact future generations [1]. The food system, environment, health of the planet and health of the population are all interconnected. The food system influences what we eat through access and availability, what we eat has health implications and environmental consequences, which in turn determines the quantity, quality, diversity, and safety of the food supply. But food systems differ around the world, and each country and region face specific environmental, socio-cultural, economic and health challenges.

There has been a vast amount of research to understand the relationships between food intake and human health and many countries have national dietary guidelines to promote population health and wellbeing [2]. More recently, there has been a significant push to better understand the impacts population food choices are having on the environment. Research has identified several synergies between diets that are better for health and better for the planet, but also that there is not always perfect alignment in achieving these goals [3, 4].

National government-endorsed food-based dietary guidelines (FBDGs) are designed to influence population dietary intake by communicating simple context- and population-specific messages about what constitutes a local healthy diet. Additionally, FBDGs are often used to inform local or national policies beyond health such as education or public procurement [5]. FBDGs have historically been written from a position of human health promotion, however the emerging interconnections between human and planetary health have led to calls to broaden their scope to address environmental sustainability in addition to human health [1, 6–8]. Some countries have adopted environmental sustainability considerations into their FBDGs [9–11] and the presence of environmental sustainability within guidelines appears to be increasing as guidelines are updated and published [12].

Global dietary guidance on healthy diets from sustainable food systems has also been published in the form of guiding principles [1] and food-based dietary targets set out in the EAT-Lancet Planetary Health Diet [7]. These guidance documents have elevated considerations within national guidelines on how dietary advice can simultaneously improve health goals for populations and the planet. However, population-level dietary change is notoriously difficult to achieve, so efforts to contextualise this guidance to specific countries, acknowledging what and how populations currently eat, is important for behaviour change at the local level. Ultimately the degree to which dietary guidance is adopted by the population will affect the health and environmental outcomes realised [13]. There are known disparities between population dietary intakes and recommendations contained within global and national dietary guidance documents. Comparisons have been made between global dietary guidance and more local dietary guidelines [14–17], and between dietary guidance and population dietary intakes [14, 18, 19]. In Australia, the average dietary intake of Australian adults and children has been compared to recommended intakes from the Australian Dietary Guidelines [20], but more comprehensive analyses of dietary patterns which relate to characteristics of healthier and more environmentally sustainable ways of eating are lacking, and to date no comparison has been made to global recommendations proposed for a healthy and sustainable diet. Therefore, the first aim of this paper was to model the EAT-Lancet Planetary Health Diet in the Australian context and compare it to the national Australian Dietary Guidelines and to the average Australian diet. This comparison focused on the food group and nutrient composition of the dietary patterns. The two benchmark sets of dietary recommendations differ in their emphasis on human health and wellbeing (the primary focus of the Australian Dietary Guidelines) and human health alongside planetary health (the focus of the EAT-Lancet Planetary Health Diet). The second aim of this paper was to compare the food group and nutrient composition of various existing dietary patterns identified within the Australian population to these benchmarks. The dietary patterns explored were selected based on single markers of perceived healthiness such as vegetable consumption, and perceived markers of environmental impact such as consumption of animal-based products, specifically meat and dairy.

## Methods

### Population dietary intake survey

The 2011–2013 Australian Health Survey was conducted by the Australian Bureau of Statistics and included the National Nutrition and Physical Activity Survey. A detailed description of the sampling framework and data collection methods of the survey is available elsewhere [21]. Briefly, data collection was conducted using a stratified multistage area sample of private dwellings. The area-based selection ensured that all sections of the population living in private dwellings within the geographic scope of the survey were represented by the sample. The survey is nationally representative, and furthermore, weighting these data prior to analysis meant the estimates reflect the demographic structure of the Australian population to infer results for the population. A detailed summary of the demographic characteristics of the Australian population and the survey sample are available online [21, 22].

As part of the National Nutrition and Physical Activity Survey trained interviewers conducted two 24-h dietary recalls. Respondents were asked to recall the previous 24-h intake of food and beverages, using a food model booklet to aid in portion size estimation [21]. Analyses were conducted using the face-to-face dietary recall (the first day of recall) which allowed for inclusion of data from the entire sample of respondents. The second day was conducted via telephone and completed by only two-thirds of respondents, reducing the sample size. There was also a significant 474 kJ difference in mean energy intake reported between day 1 and day 2 of the survey, suggesting day 2 data may be subject to additional mis- or underreporting.

Nutrient intake data were derived from the Australian Food, Supplement and Nutrient Database (AUSNUT) 2011–2013 [23] developed for the National Nutrition and Physical Activity Survey. Servings of food groups consumed were calculated using the National Nutrition and Physical Activity Survey 2011–2013 confidential unit record files Food Level Data [24]. In these data, food and beverages were disaggregated into their core food group components, and the number of servings of each food group per portion consumed provided. Discretionary choices were defined using the Discretionary Food List developed for this survey [25]. These foods and beverages are those high in added sugar, salt, saturated fat and/or alcohol. Servings of discretionary choices were calculated as 600 kJ portions, as is consistent with the Australian Dietary Guidelines [26].

### Population subgroup analysis

In Australia, the dietary guidelines and Nutrient Reference Values differ by age group [27]. The Australian Dietary Guidelines make recommendations for three adult age groups (19–50; 51–70; and 71 + years). For ease of interpretation, this analysis was limited to one age group from the dietary guidelines – those aged 19–50 years (*n* = 5,920), which was the largest adult age group, comprising 55.2% of the adult sample included in the survey. This analysis examined the average diet for adults in the 19–50 years age group, and the average diet of males and females in this age group. This analysis also examined different existing dietary patterns that were identified within the population using a priori approach. These dietary patterns were conceptualised based on current knowledge of single focused nutrition advice relating to health and environmental sustainability. For example, dietary patterns that contained lower and higher amounts of foods often considered to be environmentally intensive (animal-based sources of meat and dairy foods), and existing dietary patterns containing lower and higher amounts of foods known to be associated with the healthier diets (higher vegetable intake and lower discretionary food intake). To create these groups, adults were stratified into four subgroups based on consumption. This was done separately for meat, dairy, vegetables, and discretionary foods. Non consumers were identified, and then consumers stratified into three equal groups based on consumption. The first and last tertiles reflected those with the ‘lowest’ and ‘highest’ intakes within each gender. For example, the ‘lowest meat’ subgroup contained adults who were in the lowest tertile for meat intake among males and females aged 19–50 years; and the ‘highest vegetable’ subgroup those adults in the highest vegetable tertile meaning they consume the greatest amounts of vegetables compared to the other adults aged 19–50 years. The tertiles were created within each gender group, and then put back together, therefore, they contain equal numbers of males and females. And finally, a dietary pattern based on diet quality identified diets that were least and most compliant with the Australian Dietary Guidelines using a validated index of dietary quality [28]. As above, tertiles of diet quality were created for males and females aged 19–50 years and the highest tertile reflected those with a dietary pattern with closest alignment to the Australian Dietary Guidelines. The lowest diet quality group had an overall diet quality score of 22 out of 100, compared to 62 out of 100 for the highest diet quality group. These 13 different dietary patterns among Australian adults (See Supplementary Table 2) were compared to the recommendations within the Australian Dietary Guidelines [26] and the Planetary Health Reference Diet [7], which are described in more detail below. The discussion of results for this paper focused on the average Australian diet, and 5 selected subgroups: the lowest meat, lowest dairy, highest vegetable, highest diet quality and lowest discretionary choices dietary patterns.

### Benchmark dietary recommendations

#### Australian dietary guidelines

The Australian Dietary Guidelines (ADGs) are designed to promote health and wellbeing in the Australian population. They are built on a food modelling system [29] where a range of dietary patterns were developed that delivered the nutrient requirements set out in the Nutrient Reference Values [27] for age and gender subgroups in the Australian population. These dietary patterns considered the usual patterns of intake of Australians as well as factors such as chronic disease risk, food culture, social equity, and practicality [29]. The modelling of these dietary patterns was extensive with many variations in dietary patterns included. As a result of the modelling, the ADGs Educators Guide recommends average daily servings for each of the following five food groups: Fruit, Vegetables, Grains, Lean meats and alternatives, Dairy foods and alternatives. A daily allowance is also provided for discretionary choices and unsaturated fats and oils. Separate recommended daily serving for the five food groups are provided for age and gender subgroups of the population, and for this analysis the recommendations for the 19–50 years age group for male and females were used. The breakdown of food choices within a food group were guided by the original modelling of the ADGs as this was based on usual patterns of eating for Australians. This modelling guided the proportion of total vegetables as starchy and other vegetables; and the breakdown of meat and alternatives as red meat, other animal-based proteins, and legumes for the current analysis. The modelling of the ADGs for this project selected specific foods within a food group, such as the cut of red meat within the red meat allowance, to be as much as possible like the Planetary Health Reference Diet modelling. Therefore, this modelled version of the ADGs could be described as a dietary pattern that includes more sustainable food choices in amounts recommended by the ADGs.

#### Adaptation of the planetary health diet to the Australian context

The Planetary Health Diet provides daily food intake recommendations for a diet that was designed to “optimise human health and environmental sustainability” as described in the EAT-Lancet report [7]. The diet was designed to meet the WHO global recommendations for all nutrients other than phosphorus and copper where the United States targets were used [30]. The Planetary Health Diet takes a global focus and includes broadly global foods from eight food groups: Fruit, Vegetables, Starchy vegetables/tubers, Wholegrains, Dairy foods, Protein sources (including meat and alternatives), Added fats and Added sugars. The recommendations provide a target based on an average amount, as well as lower and upper boundaries (in grams) for each food group listed. This analysis used the Reference Diet which is based on the average value. In its development, the Planetary Health Reference Diet (PHRD) was modelled using examples of commonly consumed foods in the United States, and the nutrient composition of the diet was originally estimated using the U.S. Department of Agriculture (USDA) Foods Database, FoodData Central [31, 32].

In the present study, the PHRD modelled using the USDA database was adapted to the Australian context using foods from the AUSNUT 2011–2013 food composition database [23]. The PHRD was modelled using a single list of 35 food items. Modelling the PHRD with a series of iterations similar to the 2013 ADGs was out of scope for this paper. Rather, individual food items were selected from the AUSNUT database using the food item name and nutrient composition that was considered the closest possible match to the USDA modelled diet [32]. See Supplementary Table 1 for a comparison of foods used in the modelling. In most circumstances there were suitable options in AUSNUT. In circumstances where the USDA modelled diet used higher fat products, such as whole milk and non-lean meat (e.g. beef, ground, 15% fat), lower fat items such as reduced fat milk and low-fat meat (e.g. beef mince < 5% fat) were used to comply with the ADGs recommendations [26]. The PHRD does not contain discretionary foods or beverages like the ADGs, however, the added saturated fats and oils, and added sugar are considered discretionary and were converted to servings of discretionary choices. The nutrient and food group composition of this adapted version of the PHRD was calculated using the AUSNUT 2011–2013 food composition database.

The food group composition of both diets was described using the five food groups, unsaturated fats, and discretionary choices, as described in the 2013 Australian Dietary Guidelines ([26], See Table 1).

**Table 1:** Classification of food groups presented in this analysis

Food groups	Description and subcategories
Fruit	Fresh fruit, dried fruit and 100% fruit juice
Vegetables	All vegetables excluding legumes Included 2 subcategories: • Starchy vegetables including white potato, sweet potato and corn • Other vegetables including leafy greens, salad and cooked vegetables
Dairy and alternatives	Milk, yoghurt, cheese and/or other alternatives
Cereals	All bread, breakfast cereal, rice, pasta and other grain products Included 2 subcategories: • Wholegrains • Refined grains
Meat and alternatives	All lean meats, poultry, fish and seafood, eggs, legumes and tofu, and nuts Included 3 subcategories: • Red meat including beef, lamb and pork^*^ • Other animal-based protein-rich foods including poultry, fish and seafood, and eggs • Other plant-based protein rich foods including legumes, tofu, and nuts and seeds^**^
Discretionary choices	Foods and beverages high in added sugar, salt, saturated fat and alcohol. For example, cakes, biscuits, pastries, pies, takeaway foods, fried potato products, sugar sweetened beverages, alcoholic beverages
Unsaturated fats and oils	All unsaturated oils, and spreads

^*^Red meat sub-category includes beef, lamb and pork as per the definition of the ADGs

^**^In the ADGs legumes are included in the vegetables category (as a 75 g serving) as well as the meat and alternatives category (as a 150 g serving). For this analysis they were considered a meat alternative. Nuts and seeds are included in both the meat and alternatives food group (as a 30 g serving) and the unsaturated fats group (as a 10 g serving). For the present analysis nuts and seeds were included as a meat alternative

### Environmental data

Environmental data derived from life cycle assessment for individual foods within the Australian food system were obtained from previous studies [33–36]. A combined index of environmental impact was used as an indication of the environmental impact of diets which included indicators of climate footprint [34], water scarcity footprint [35], and cropland scarcity footprint [36]. The environmental impact data for individual foods consumed were summed to estimate the environmental impact of individuals’ diets.

### Statistical analysis

Statistical analysis was performed using the IBM SPSS statistical package version 25 [37]. Summary estimates were weighted to reflect the demographic structure of the Australian population using weights based on age, gender, and residential area. An additional weighting factor was applied to correct for the day of the week of the survey. The percentage of subjects reporting their intake for Saturday (3.5%) and to a lesser extent Friday (11.4%) was underrepresented compared with the expected percentage of 14.3%. Therefore, the data presented were weighted using the ABS population weighting and the day of the week weighting.

Estimated mean food and nutrient intakes of the identified dietary pattern groups are presented and were based on one day of diet recalls and represent the mean usual intake of the group, not usual intake of an individual. Food group composition of the dietary patterns identified were compared to those in the modelled ADGs diet and those modelled from the adapted PHRD. The average nutrient composition of the dietary patterns was compared to the appropriate Nutrient Reference Values for Australia. The mean nutrient composition of the diets was expressed as a percentage of the Nutrient Reference Values for males and females separately, and the average percentage presented.

## Results

### Food group composition of guidelines for healthy and sustainable diets

Table 2 shows the food group composition of the diets modelled on the ADGs and the PHRD which were the two benchmark dietary patterns against which current Australian diets were compared. The modelled ADGs diet included more vegetables (5.50 vs 3.83 servings), fruit (2.00 vs 1.33 servings), dairy and alternatives (2.50 vs 0.96 servings) and discretionary foods (2.75 vs 1.49 servings) than the PHRD. In contrast, the PHRD included more cereals (7.63 vs 6.00 servings) driven by more refined grains (3.87 vs 2.13 servings). The PHRD also included more total servings from the meat and alternatives food group (4.05 vs 2.75 servings) because of a much higher recommendation for plant-based alternatives which included legumes and nuts (3.26 of 4.05 total servings vs 1.35 of 2.75 total meat and alternative servings). The servings of unsaturated fats were also higher in the PHRD than the ADGs (Table 2).

**Table 2:** Comparison of recommended number of servings of food groups in the Australian Dietary Guidelines, the EAT Lancet Planetary Health Reference Diet and the average Australian diet (adults 19–50 years)

	**Australian Dietary Guidelines**	**Planetary Health Reference Diet**	**Average Australian Diet Adults 19–50 years**
VEGETABLES	5.50	3.83	2.72
Starchy veg	1.13	0.44	0.55
Other veg	4.37	3.39	2.18
FRUIT	2.00	1.33	1.44
DAIRY & ALT	2.50	0.96	1.55
CEREALS	6.00	7.63	4.87
Wholegrains	3.87	3.76	1.41
Refined grains	2.13	3.87	3.46
MEAT & ALT	2.75	4.05	2.31
Red meat	0.70	0.15	1.01
Animal-based alt	0.70	0.63	1.00
Poultry	0.23	0.25	0.68
Fish seafood	0.23	0.28	0.19
Eggs	0.23	0.10	0.12
Plant-based alternatives	1.35	3.26	0.30
Legumes	0.63	1.59	0.08
Nuts	0.72	1.67	0.22
UNSATURATED FATS	4.00	5.71	2.24
DISCRETIONARY CHOICES	2.75	1.49	5.57

### Comparison of the average Australian adult diet to guidelines

**Fig. 1:**
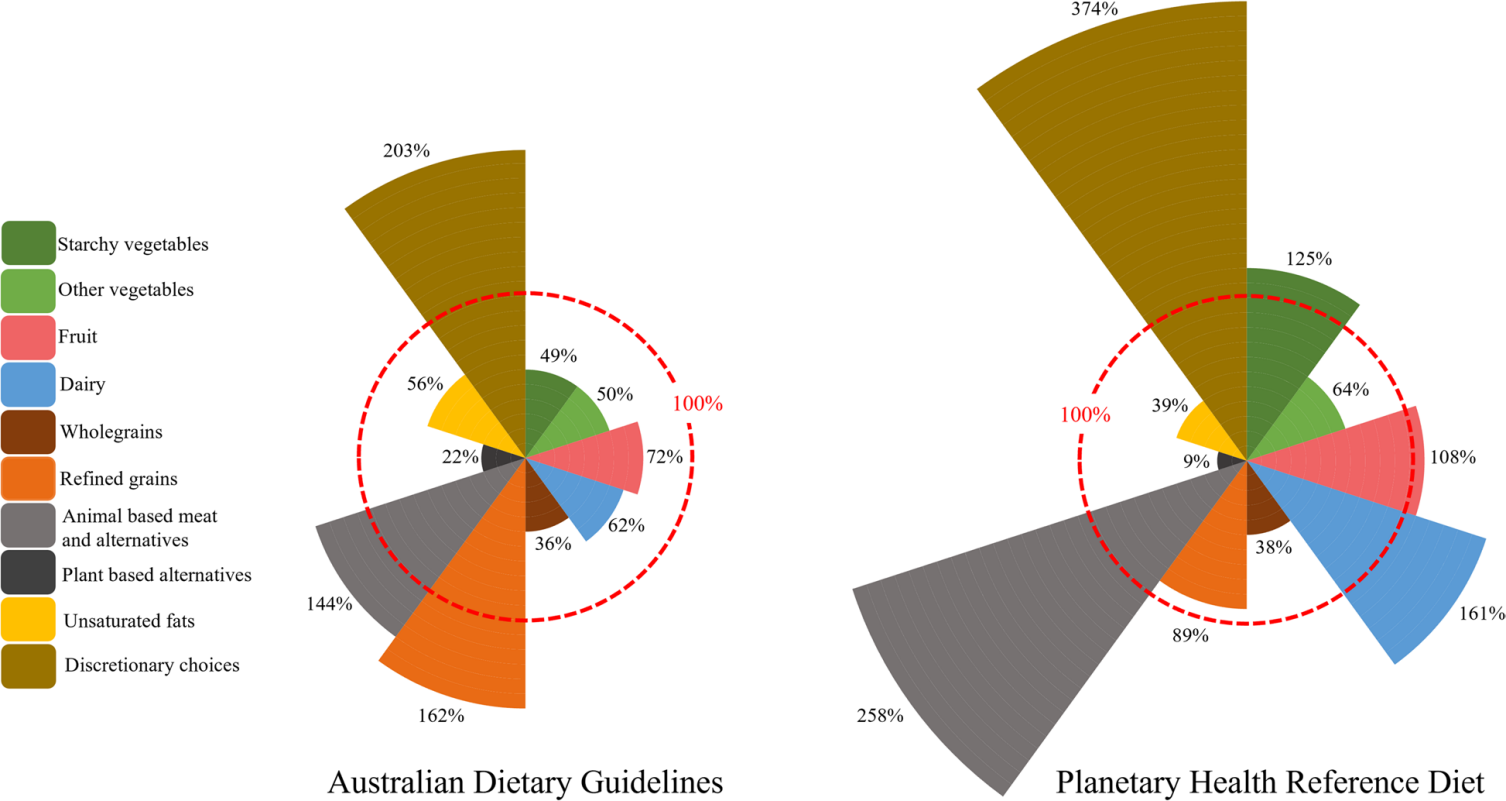
Comparison of the average Australian diet (adults 19–50 years) with the 2013 Australian Dietary Guidelines and the EAT Lancet Planetary Health Reference Diet. The average Australia diet is expressed as a percentage of the benchmark recommendations. The red dashed line represents 100% of the recommendations in the Australian Dietary Guidelines or the Planetary Health Reference Diet

Figure 1 is a visual comparison of the average diet of Australian adults aged 19–50 years to the modelled ADGs and PHRD, expressed as a percentage of the benchmark. The average Australian diet contained less dairy and alternatives (1.55 servings, 62% of ADGs), less unsaturated fats (2.24 servings, 56% of ADGs), less fruit (1.44 servings, 72% of ADGs) and about half as many vegetables (2.72 vs 5.50 servings, 49% of ADGs) as the ADGs. Overall, the average diet was also lower in cereal foods than the ADGs (4.87 vs 6.00 servings); however, disaggregating this food group showed the average diet was lower in wholegrains (36%) but higher in refined grains (162%) than the ADGs. Likewise, disaggregating the meat and alternative food group showed the amounts of red meat and other animal-based alternatives in the average diet was similar to the ADGs (within a third of a serving), but the ADGs contained 1.35 servings of plant-based alternatives compared to 0.30 servings in the average diet (equivalent to 22% of ADGs) (Fig. 1).

The overall recommendations for meat and alternatives in the PHRD exceeded the ADGs because of the inclusion of 3.26 servings of plant-based alternatives. The PHRD recommends small amounts of animal-based meat and alternatives, and so the average Australian diet contain more than twice as much animal-based meat and alternatives than the PHRD (258%). Interestingly, the PHRD and the ADGs recommend similar amounts of poultry and seafood. The average Australian diet contained similar amounts of fruit as the PHRD, but almost four times more discretionary foods (5.57 vs 1.49 servings, 374%) and about 1.5 times more dairy and alternatives (1.55 vs 0.96 servings, 161%). Despite exceeding the PHRD recommendation, dairy and alternatives consumption of the average Australian diet was below ADGs recommendation (Fig. 1).

### Composition of selected Australian diets compared to guidelines

**Fig. 2:**
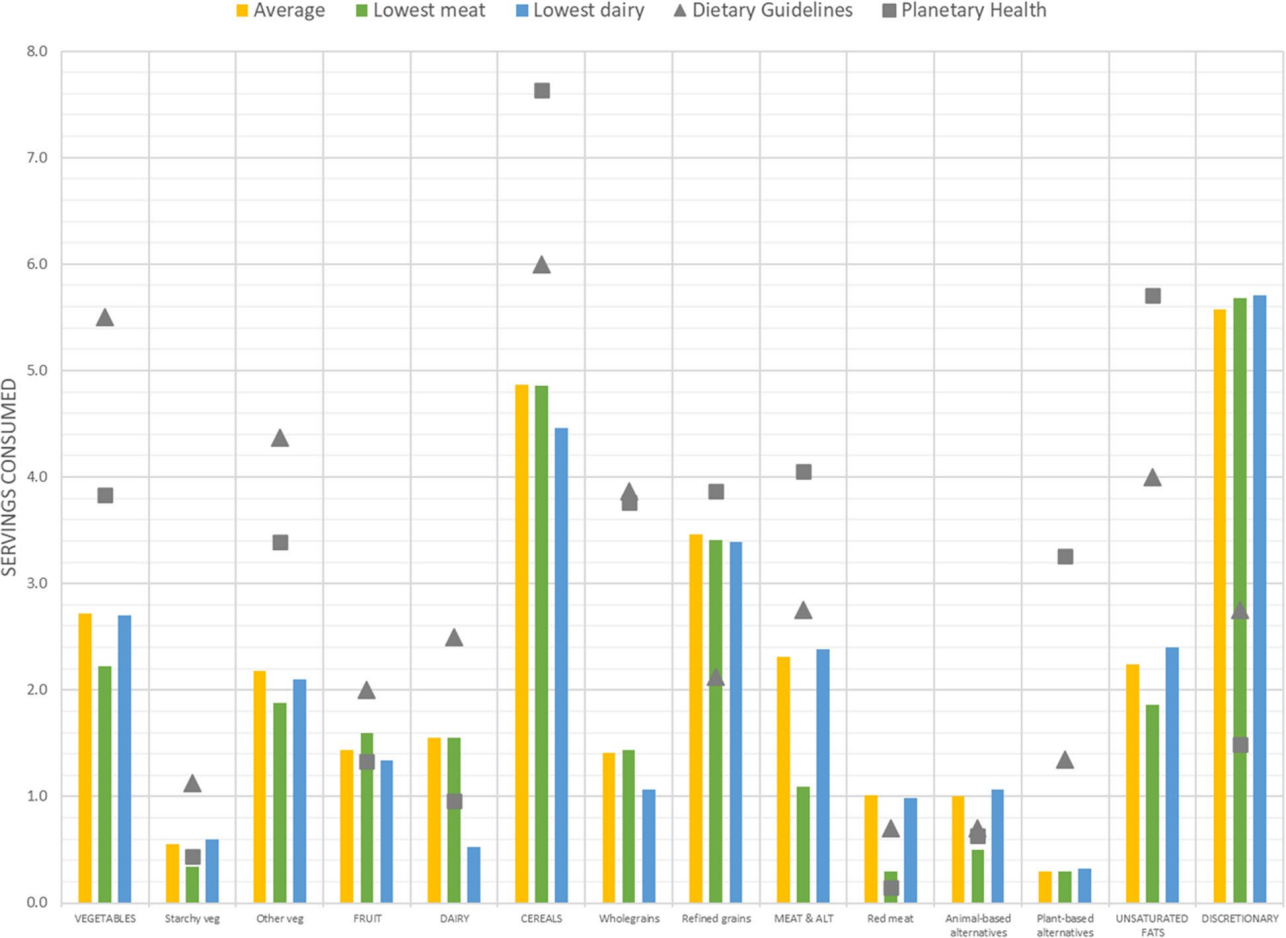
Daily average food group intake (servings per day) of selected Australian adults aged 19–50 years and stratified by levels of meat and dairy consumption and compared to the Australian Dietary Guidelines and the Planetary Health Reference Diet. Values are presented as means

Dietary patterns within the population can vary substantially, which is not reflected when examining the average pattern. To understand the degree to which various diets within the Australian population aligned with the modelled ADGs diet and the PHRD, we examined selected dietary patterns based on gender, level of consumption of meat and dairy foods, level of consumption of vegetables and discretionary foods, and overall diet quality (Supplementary Table 2). Figure 2 shows the food group composition of the average Australian diet, as well as the composition of the diets of a subgroup of Australians with the lowest consumption of animal-based meat and dairy foods and compared these to the modelled ADGs diet and the PHRD. A diet that was lowest in animal-based meat contained 1.09 servings of the meat and alternatives food group. The amount of red meat in this dietary pattern (0.30 servings) was one third of the average Australian diet (1.01 servings), and about half of that recommended in the ADGs (0.70 servings), but twice that recommended in the PHRD (0.30 vs 0.15 servings). The amount of other animal-based alternatives (poultry, fish and seafood, eggs) was slightly higher in the PHRD than the lowest meat pattern (0.63 vs 0.50 servings) and plant-based alternatives substantially higher (3.26 vs 0.30 servings).

Intake of dairy and alternatives was 0.53 servings and 3.21 servings among Australian adults with the lowest and highest consumption respectively (Supplementary Table 2). Intake of dairy and alternatives for the lowest subgroup of Australian consumers (0.53 servings) was about half the amount recommended in the PHRD and about 20% of the recommend amount in the ADGs (0.96 and 2.50 servings respectively). Similar to the diets with lowest meat, the diets lowest in the dairy and alternatives food group were lower in vegetables, wholegrains, and unsaturated fats than the modelled ADGs diet and PHRD and exceeded the recommended amounts of discretionary foods.

**Fig. 3:**
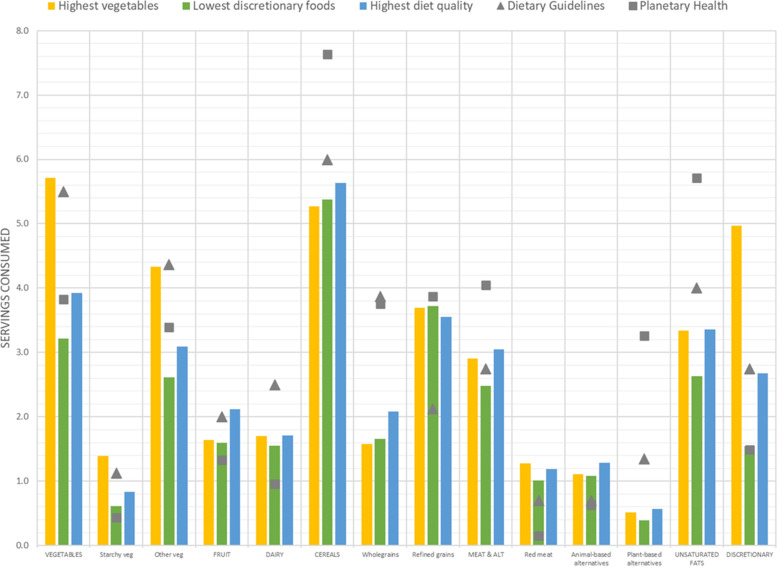
Daily average food group intake (servings per day) of selected Australian adults aged 19–50 years stratified by indicators of a healthy diet (highest vegetables, diet quality and lowest discretionary choices) and compared to the Australian Dietary Guidelines and the Planetary Health Reference Diet. Values are presented as means

Figure 3 shows three selected dietary patterns developed based on markers of healthiness. Vegetable intake in the population subgroup with the highest level of consumption was 5.71 servings per day, which was similar to the ADGs and about 2 servings higher than the PHRD (Supplementary Table 2). This dietary pattern was also similar to the ADGs recommended pattern in terms of the amount of fruit, meat and alternatives, and unsaturated fats. However, it was lower in dairy and alternatives and cereal foods, and higher in discretionary foods than the ADGs recommended pattern. The Australian diets with the lowest amounts of discretionary foods, did not necessarily contain adequate amounts of the healthy five food groups. These diets contained less vegetables (3.22 vs 5.50 servings), dairy and alternatives (1.55 vs 2.50 servings), wholegrains (1.66 vs 3.87 servings), and unsaturated fats and oils (2.63 vs 4.00 servings) than the ADGs diet, and less wholegrains (1.66 vs 3.76 servings), meat and alternatives due to less plant-based alternatives (0.39 vs 3.26 servings) and unsaturated fats (2.63 vs 5.71 servings) than the PHRD. The diet with the lowest intake of discretionary choices also contained more dairy and alternatives (1.55 vs 0.96 servings) and red meat (1.01 vs 0.15 servings) than the PHRD.

Because diet quality was operationalised as compliance with ADGs, the food group consumption of the subgroup of the population with the highest diet quality was most closely aligned with this set of guidelines. The diets of this subgroup still consumed less vegetables (3.92 vs 5.50 servings) and less dairy and alternatives (1.71 vs 2.50 servings) than the modelled ADGs. None of the dietary patterns of the subgroups examined in this analysis consumed cereals, plant-based meat alternatives to meat (legumes and nuts) and unsaturated fats in amounts close to the recommendations in the PHRD.

### Types of meat and alternatives within selected dietary patterns

**Fig. 4:**
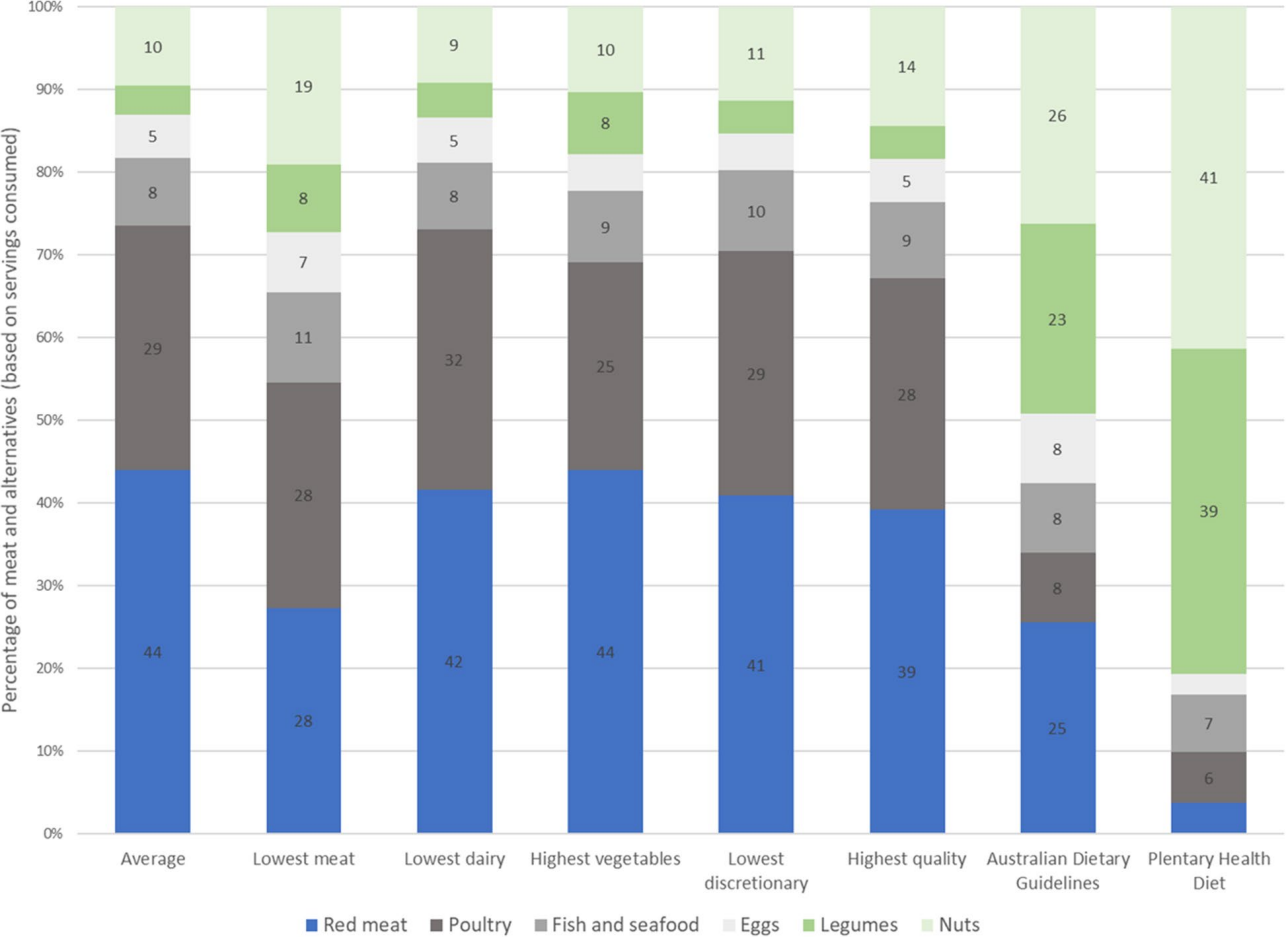
Daily average consumption of red meat, animal-based alternatives (broken down into subcategories of poultry, fish and seafood, eggs) and plant-based alternatives (legumes, tofu and nuts and seeds) as a percentage of total meat and alternatives and compared to the Australian Dietary Guidelines modelled diet and the Planetary Health Reference Diet. Values are presented as a percentage. *Data values are not presented when the percentage was less than 5%

Figure 4 shows the breakdown of the types of meat and alternatives as a proportion of total servings. Typically, in Australian diets, irrespective of the amount of meat, vegetables, discretionary foods, or overall quality, the proportion of total meat and alternatives consumed as red meat was between 28 and 44%, and for poultry between 25 and 32% of the total meat and alternatives This compared to 25% red meat and 8% poultry in the modelled ADGs, and 4% red meat and 6% poultry in the PHRD. It is recommended that when following the PHRD, most servings (around 80%) selected from the meat and alternatives food group should be plant-based, such as legumes and nuts. Among Australian diets, the subgroup with the lowest meat consumption had the highest proportion as plant-based alternatives (27%), followed by those with the highest diet quality (18%) and highest vegetable consumption (18%). Proportionally, fish and seafood contributed similar amounts to total meat and alternatives in the ADGs, PHRD and average Australian diet (~ 7–8%).

### Nutrient adequacy of selected dietary patterns

The estimated energy intake of the average diet of Australians aged 19–50 years was 9191 kJ, compared to 11421 kJ for the modelled ADGs diet and 10242 kJ for the modelled PHRD (Table 3). The following analysis is a comparison of the nutrient composition of the selected dietary patterns to the Australian nutrient recommendations; the Estimated Average Requirements (EAR) or the Adequate Intake (where no EAR was available). Sodium intake was compared with the Suggested Dietary Target (SDT). For the 22 nutrients examined, intake was compared with the appropriate nutrient reference value and expressed as a percent.

**Table 3:** Estimated environmental impact and daily average nutrient intake of Australian adults aged 19–50 years stratified by levels of meat and dairy intake, and indicators of a healthy diet (highest vegetable, lowest discretionary food and beverage consumption, and highest overall diet quality) and compared to the Australian Dietary Guidelines and the Planetary Health Reference Diet. Values are presented as kilojoules for energy and as a percentage of the nutrient reference value for all other nutrients

	**Average diet (9,191 kJ)**	**Lowest meat (8,364 kJ)**	**Lowest dairy (8,499 kJ)**	**Highest vegetables (10,252 kJ)**	**Lowest discretionary (7,178 kJ)**	**Highest quality (8,912 kJ)**	**Dietary Guidelines (11,421 kJ)**	**Planetary Health (10,242 kJ)**
Protein EAR	191	145	171	219	177	212	195	178
Dietary Fibre AI	82	81	77	113	83	103	143	139
Thiamin EAR	165	158	146	186	159	185	191	196
Riboflavin EAR	197	183	144	223	184	220	224	148
Niacin EAR	374	298	347	425	347	408	198	190
VitB6 EAR	148	122	134	177	133	155	202	199
Vit B12 EAR	234	180	175	255	208	247	241	135
Folate EAR	191	189	164	218	185	210	225	200
Vit A EAR	143	125	127	243	147	188	245	194
Vit C EAR	345	324	340	502	347	438	554	420
Calcium EAR	101	98	63	116	94	110	123	71
Phosphorus EAR	262	221	219	302	235	284	337	288
Zinc EAR	128	97	111	153	120	145	135	126
Iron EAR	169	147	148	208	159	190	220	234
Magnesium EAR	116	105	101	142	110	135	200	199
Iodine EAR	179	170	137	191	160	183	208	116
Selenium EAR	170	135	165	193	160	192	119	125
Sodium SDT	130	121	119	143	102	113	18	11
Potassium AI	100	87	87	132	93	116	166	140
Linoleic acid AI	100	87	98	114	82	105	155	236
Alpha-linolenic acid AI	141	122	138	166	118	148	154	231
Omega 3 AI	219	138	201	252	259	308	376	418
Total number of nutrients met (out of 22)^*^	18	16	17	21	17	21	22	21
Estimated environmental impact	0.13	0.10	0.11	0.14	0.11	0.13	0.09	0.07
Estimated environmental impact (adjusted to be per 10,000kj)	0.14	0.12	0.13	0.14	0.15	0.15	0.08	0.07

^*^Values greater than 100% reflect meeting or exceeding the Nutrient Reference Value and was considered the beneficial direction for all nutrients except sodium where a value less than 100% was considered the beneficial direction

The ADGs, as designed, provided more than 100% of the EAR or AI for all the nutrients assessed, and among the selected diets, the highest diet quality pattern, being the one closest to the ADGs recommendations, met the nutrient reference values for 21 out of 22 nutrients examined. Like all the selected dietary patterns, the highest quality diet exceeded the SDT for sodium.

The PHRD also met 21 of the 22 for the nutrients assessed, falling short of the EARs for calcium (71% of EAR). Selected diets developed based on lower or higher intake of one food group tended to be most at risk of not meeting the nutrient reference values. The lowest meat and lowest dairy patterns met the EARs for 16 and 17 of the 22 nutrients assessed, respectively, with dietary fibre, calcium, potassium, linoleic acid below the EAR and sodium exceeding the SDT for both dietary patterns. Zinc was also below the EAR for the lowest meat diet. The nutrients most commonly at risk of insufficiency in the selected Australian dietary pattens were dietary fibre, calcium, potassium, and linoleic acid, with sodium at risk of excessive intakes.

### Environmental impacts of selected dietary patterns

The combined environmental index value for the average Australian diet was 0.13 (Table 3). The environmental impact value for the ADGs modelled using foods with lower environmental impacts was 31% lower, and the PHRD 46% lower than the average Australian diet. Among the selected Australian diets examined here the diets with least meat and dairy had the lowest environmental impacts.

Given there is a relationship between dietary energy and dietary environmental impacts, comparing diets of similar kilojoules can highlight the impact of food choices on dietary environmental impacts. When the environmental impact index value for each diet was adjusted to a standardised 10,000 kJ, the rank order of the diets changed slightly, however the modelled ADGs diet and the PHRD still had the lowest environmental impacts (Table 3). That said, iso-caloric comparisons need to be interpreted with care since food choice and total energy intake are not independent.

## Discussion

This paper compared the daily food group recommendations contained within in the Planetary Health Reference Diet (PHRD), a global set of dietary recommendations, to those set out in the Australian Dietary Guidelines (ADGs) Educator Guide and various dietary patterns exhibited by the Australian population. The PHRD was found to differ from Australia’s national dietary guidelines considerably in relation to the proportion of the diet provided by the meat and alternatives food group. This difference was due to the higher suggested intake of plant-based alternatives including legumes and nuts which made up 80% of the total amount of meat and alternatives recommended. The PHRD does not include discretionary choices but rather contains an allowance for saturated fats and added sugar. When converted to servings of discretionary foods, the ADGs contained about twice as many servings of discretionary foods as the PHRD. The environmental index impact values for these two benchmark diets were 31–46% lower than for the typical Australian diet. The comparison of selected dietary patterns among Australians to a set of global and national benchmarks in terms of food group composition, nutrient adequacy and environmental impacts provides useful insights into the complexities of population level nutrition advice to improve the health of our people and the planet.

The role of national dietary guidelines is to support their population to make healthier food choices, in a balance that promotes human health and more recently environmental sustainability [1, 6–8]. However, this analysis showed that the average Australian adult diet contained at least one less serving of vegetables, dairy foods, grains and in particular wholegrains, and unsaturated fats than what is recommended for health and wellbeing in the Australian national dietary guidelines. Some subgroups of the population, such as those adults with the highest vegetable intake, did achieve the daily recommendations for vegetables, however these adults did not necessarily consume the recommended amounts for all the other food groups that form part of a healthy diet according to the ADGs. Likewise, those with the lowest intake of discretionary foods and beverages did not necessarily consume adequate amounts of the healthy five food groups. Animal-based food groups such as dairy and meat, and in particular red meat, have higher environmental impacts per serving compared to other foods [34]. While Australian diets lowest in these foods had lower environmental impacts than other selected Australian diets, the overall consumption was below many of the food group recommendations for health, and subsequently these diets were among the least nutritionally adequate dietary patterns analysed here. Focusing on single food groups or nutrients has been the basis of some nutrition advice in the past, however, there have been calls to move away from this reductionist approach towards more of a whole-of-diet or dietary pattern approach [38–40] and our results support this. The ‘highest diet quality’ pattern was closest to the ADGs recommendations without necessarily achieving full compliance with the guidelines (average score 62 out 100). So, while not necessarily ideal or the healthiest possible diet, it was pleasing that this pattern achieved the estimated requirements for all nutrients examined, except sodium. Importantly this dietary pattern provides an example of an actual way some Australians are eating (1,928/5,920; 33% of adults 19–50 years), which if adopted by more people, could result in improvements in diet quality and health. However, the estimated environmental impact of this pattern of eating was similar to that of the average Australian diet, so further changes in food choices within food groups would be required in order to achieve improved healthiness and reduced environmental impacts compared with the average Australian dietary pattern.

There is consensus that diets need to change but achieving significant change has been a long-standing public health challenge. There are also growing calls for transformation of the food system [41] so that everyone can eat a nutritious diet of healthy foods produced from a food system that is environmentally, socially, and economically sustainable [41, 42]. It is also a challenge to balance public health and individual nutrition goals with environmental objectives [42]. One starting point for change is in dietary guidelines and ensuring these documents and their advice acknowledges the connections between the dimensions of food, health and the environment [39]. The PHRD provides a set of food intake targets for a diet that was promoted as healthy for both people and the planet. The Planetary Health pattern of eating includes more plant-based foods and fewer animal-based foods than currently consumed in Australia (and most developed countries) and as such, almost all the selected Australian diets fell well short of the PHRD recommendations for vegetables, wholegrains, legumes and nuts, and were well above recommendations for dairy foods, red meat, and discretionary foods (some of which contain animal-sourced ingredients). The PHRD modelled in the Australian context provided inadequate amounts of calcium (71% of the EAR). Prevalence of inadequate consumption of calcium in Australia is already high, with 73% of females and 51% of males aged two year and over consuming less than the calcium EAR [43]. Therefore, the PHRD dietary pattern may pose further risks for bone health if adopted. However, the PHRD was the diet with the lowest estimated environmental impacts so among the diets modelled here could be considered the healthiest for the planet.

The emphasis on consuming a wide variety of foods within the current ADGs is sometimes overlooked but re-emphasising the statements around promoting a variety of “different types and colours” of vegetables and “the wide variety of foods” within the meat and alternatives group would be important for human and planetary health in contemporary dietary guidance. The average Australian diet does not exceed the recommended number of servings of the meat and alternatives food group set out in the ADGs or the PHRD, but the proportion of animal-based to plant-based servings does not align with either set of guidelines. Australians consume 73% of servings from the meat and alternatives food group as red meat and poultry. Whereas the ADGs suggest about one third of servings from this food group come from red meat and poultry. This ADGs recommendation, therefore, falls between current intakes (73%) and the PHRD (10% of the meat and alternatives food group to be consumed as red meat and poultry). Diversifying food choices within the meats and alternatives food group, and all food groups for that matter, is recommended. However, this would require significant dietary change for most Australians (intake of legumes would need to increase by about 20-fold). Substantially increasing legume consumption would be a big cultural shift for Australians, as they are not commonly part of meals [44]. Reductions in animal meats can make it more difficult to achieve adequate iron and zinc intakes so careful meal planning is required, particularly as the bioavailability of these nutrients is lower in plant-based foods. Further, switching to some highly processed plant-based meat alternatives may not have the assumed health benefits [45–47], and other plant-based alternatives such as nuts may also come at a higher environmental cost depending on local environmental constraints [35]. Therefore, developing dietary advice can be difficult as there are trade-offs between food, health and the environment that need consideration.

Consistent across all of the selected Australian diets examined was a pattern of eating that exceeded both the global and national recommendations for discretionary foods and beverages, which are those energy dense, nutrient poor items high in sodium, added sugar, and saturated fat, that when consumed in excessive amount can increase the risk of weight gain and obesity [48, 49]. The average Australian diet contained about twice the national recommended maximum intake for discretionary foods and beverages. The widespread overconsumption of discretionary foods means it is increasingly important to examine their impacts on the healthiness of diets, and in turn their environmental impacts [50]. Excessive consumption of discretionary choices leaves insufficient room in the diet for the five healthy food group foods. Evidence is emerging around the health implications of overconsuming ultra-processed foods [48, 51] and evidence is also starting to emerge around the environmental impacts of discretionary, ultra-processed foods and beverages. Discretionary foods and beverages have been estimated to account for 28–34% of diet-related climate [34], water [35], cropland [36] and pesticide [52] footprints of the Australian diets. It is also important to consider the opportunity cost of processing and consuming these foods. Dietary guidelines suggest they are not a necessary part of the diet as they may displace healthier foods, but they do have a place in a healthy diet for variety and enjoyment [26], so again finding the balance around enjoyment, health and environmental sustainability is important.

This study used data from a nationally representative sample of Australians through which dietary intake was assessed using a robust 24-h recall methodology and the sampling framework of the survey allowed for inferences about the population. However, dietary survey data using 24-h recall are likely to under report true dietary intake based on analysis of energy requirements [21, 53], and misreporting may occur differentially between different food groups, which may have influenced results. For example, it is known that individuals tend to overreport their intake of healthy foods such as vegetables and underestimate their intake of less desirable foods such as those within the discretionary food and beverage category [21]. One 24-hour recall was used to assess usual group intake because it maximized the number of participants included (only 64% of participants completed a second recall, and by telephone). The reported individual intakes represent food group intake on a day, not usual intake, however the mean of the individual intakes for groups is a good representation of mean usual intake [54]. The study selected a range of a priori dietary patterns considering single markers of healthiness or environmental impact. There are limitations to this approach, and the authors acknowledge that others dietary patterns could have been selected and different comparisons made. Likewise, other valid approaches to identifying the dietary patterns of interest could also have been used, such as taking a statistical approach such as using cluster analysis to identify existing dietary patterns in the population [55]. Likewise, the environmental impacts of dietary patterns are impacted by food choices and the amounts consumed. The ADGs was modelled using similar foods to the PHRD which aren’t necessarily aligned to the population’s preferences. Our other research has suggested when modelled using current food choices, the ADGs have a higher environmental impact than the average diet due to increases in vegetables and dairy required to meet the recommendations [34]. Definitions used in the study are also important and may have influenced the results. For example, these results found that the average intake of lean meat by Australians did not exceed the national dietary guidelines, however if meat consumed from all sources including discretionary sources such as sausages rolls, pies and processed meats were included then intake would likely exceed the recommendations provided in the dietary guidelines, and other guidelines for disease prevention [56, 57].

Food-based dietary guidelines are designed to be reviewed and updated based on the latest evidence of diet-disease relationships, so they are not a static benchmark even though the broad messages have been consistent over time. The urgency and interest in moving towards a sustainable food system has resulted in a greater inclusion of environmental sustainability into guidelines around the world [8, 12], as well as understanding the inclusion of sustainability more broadly to consider economic, sociocultural and political domain dimensions of sustainability with environment and human health [8]. This study compared the ADGs, which were last updated in 2013, to a more recent set of recommendations in the PHRD published in 2019. The differences in the recommended intake of the various food groups between the ADGs and the PHRD might be expected given the scientific evidence around environmentally sustainable diets has increased in the time between the publication of these documents, and future revisions may see changes to food group recommendations for Australians to reflect evolving evidence from both health and environmental science disciplines. This study only modelled one set of foods for the PHRD. While it highlighted that the PHRD failed to meet the calcium Nutrient Reference Values set for Australia and New Zealand, other nutrient recommendations were met. It is possible that other combinations of foods, or more complex modelling using multiple iterations, like that was used in the original development of the ADGs, may have resulted in a more nutritionally adequate dietary pattern, however complexity in the modelling was out of scope for this study. The study also only modelled diets for an adult subgroup (men and women 19–50 years) and different dietary risks may be identified in other subgroups, such as children or older adults.

The results of this paper have been primarily discussed in the context of the Australian population changing their dietary intake, but it is important to note that the influence of dietary change alone is limited [34]. Change in diet can help to build demand for healthy and more sustainably produced food, but the production and manufacturing of food and the amount of food that is wasted or lost during production can also be important points of intervention as these too can have a positive impact on human health and environmental sustainability of the entire food system [34, 58].

## Conclusions

Global dietary guidelines may well influence the revision of national dietary guidelines, but a country’s dietary guidelines are designed to better support dietary change as they are more considerate of local culture and practices. Local guidelines are therefore thought to be more achievable and feasible for adoption by the intended population, however in this analysis the average Australian diet fell short of many recommendations in the ADGs. Some of the messages contained within the current ADGs are consistent with the recommendations of Planetary Health Diet, but some differ considerably. Regardless of the set of guidelines referred as the benchmark, Australian diets contained suboptimal amounts of foods from the five food groups, including vegetables, wholegrains, and plant-based protein sources and they exceed the maximum recommended intake of discretionary foods and beverages. Significant behaviour change is required to improve the healthiness and environmental sustainability of population dietary habits in Australia, and these results provide a deeper understanding of areas that are furthest from recommendations.

## Supplementary Information

**Additional file 1:** **SupplementaryTable 1.** List of foods used in the development of the Planetary Healthy Dietusing the USDA FoodData Central and the comparison food used to model thePlanetary Health Diet for the Australian context using the FSANZ AUSNUT2011-2013 Food Composition databases [22]. **SupplementaryTable 2.** Daily average food group intake (servings per day) of Australianadults aged 19-50 years stratified by gender, levels of meat and dairy intake,and indicators of a healthy diet (highest vegetable, lowest discretionary foodand beverage consumption, and highest overall diet quality) and compared to theAustralian Dietary Guidelines and the Planetary Health Reference Diet. Valuesare presented as means. 

## Data Availability

The dietary intake data are publicly available from the Australian Bureau of Statistics (http://www.abs.gov.au/australianhealthsurvey accessed on 15 March 2017). The additional dataset used for this analysis are available from the corresponding author on reasonable request.

## Abbreviations

ADGs: Australian Dietary Guidelines
PHRD: Planetary Health Reference Diet
USDA: United States Department of Agriculture
