# Correction: Towards healthier and more sustainable diets in the Australian context: comparison of current diets with the Australian dietary guidelines and the EAT-lancet planetary health diet

**DOI:** 10.1186/s12889-026-28012-w

**Published:** 2026-06-16

**Authors:** Gilly Hendrie, Megan A. Rebuli, Genevieve James‑Martin, Danielle L. Baird, Jessica R. Bogard, Anita S. Lawrence, Bradley Ridoutt

**Affiliations:** 1 CSIRO Health and Biosecurity, Adelaide, South Australia Australia; 2 CSIRO Agriculture and Food, St Lucia, QLD Australia; 3 University of Melbourne, Parkville, VIC Australia; 4 CSIRO Agriculture and Food, Clayton South, Victoria Australia

**Correction: BMC Public Health 22**,** 1939 (2022)**

**https://doi.org/10.1186/s12889-022-14252-z**

Following publication of the original article [1], the authors would like to reverse the order of the “Planetary Health Reference Diet and Australian Dietary Guidelines” to correctly align with the sequence of the percentages within the sentence.

The sentence currently reads:

The environmental impact scores of the **Planetary Health Reference Diet and Australian Dietary Guidelines** were 31% and 46% lower than the average Australian diet.

The sentence should read:

The environmental impact scores of the **Australian Dietary Guidelines and Planetary Health Reference Diet** were 31% and 46% lower than the average Australian diet.

The original article [1] has been updated.
