# Supplementary material for: Towards healthier and more sustainable diets in the Australian context: comparison of current diets with the Australian Dietary Guidelines and the EAT-Lancet Planetary Health Diet
Source: BMC Public Health. 2022 Oct 19;22:1939. doi: 10.1186/s12889-022-14252-z (PMC9583557; doi:10.1186/s12889-022-14252-z)
Supplement: Supplementary file 1 — Additional file 1: SupplementaryTable 1. List of foods used in the development of the Planetary Healthy Dietusing the USDA FoodData Central and the comparison food used to model thePlanetary Health Diet for the Australian context using the FSANZ AUSNUT2011-2013 Food Composition databases [22]. SupplementaryTable 2. Daily average food group intake (servings per day) of Australianadults aged 19-50 years stratified by gender, levels of meat and dairy intake,and indicators of a healthy diet (highest vegetable, lowest discretionary foodand beverage consumption, and highest overall diet quality) and compared to theAustralian Dietary Guidelines and the Planetary Health Reference Diet. Valuesare presented as means. [file 12889_2022_14252_MOESM1_ESM.docx]

Supplementary Table 1: List of foods used in the development of the Planetary Healthy Diet using the USDA FoodData Central and the comparison food used to model the Planetary Health Diet for the Australian context using the FSANZ AUSNUT 2011-2013 Food Composition databases (22).

| United States of America: USDA Food Central Foods | AUSTRALIA: FSANZ AUSNUT 2011-13 Foods |
| --- | --- |
| Wheat, hard red spring | Flour, wheat, white, plain |
| Rice, brown, long-grain, raw | Rice, brown, uncooked |
| Potato, flesh and skin, raw | Potato, pale skin, unpeeled, raw |
| Raw spinach | Spinach, fresh, raw |
| Baked, unsalted winter squash (yel.sqs) | Pumpkin, butternut, peeled, fresh or frozen, baked, roasted, fried, stir-fried, grilled or BBQ'd, no added fat |
| Boiled, drained, unsalted carrots | Carrot, mature, peeled or unpeeled, fresh or frozen, boiled, microwaved or steamed, drained |
| Ripe, red tomatoes | Tomato, common, raw |
| Raw onions | Onion, mature, peeled, fresh or frozen, raw, not further defined |
| Boiled, drained, unsalted summer squash (zuke) | Zucchini, green skin, fresh or frozen, peeled or unpeeled, boiled, microwaved or steamed, drained |
| Raw green peppers | Capsicum, green, fresh or frozen, raw |
| Raw apples with skin | Apple, unpeeled, raw, not further defined |
| Raw oranges | Orange, peeled, raw, not further defined |
| Raw bananas | Banana, cavendish, peeled, raw |
| Whole milk (3.25%) | Milk, cow, fluid, reduced fat (1%) |
| Lentils, raw | Lentil, dried |
| Beans, navy, mature seeds, raw | Bean, haricot, dried |
| Peanuts, all types, raw | Nut, peanut, with skin, raw or dry roasted, unsalted |
| Oil roasted, unsalted almonds | Nut, almond, with or without skin, roasted, unsalted |
| Oil roasted, unsalted cashews | Nut, cashew, roasted, unsalted |
| Soybeans, mature seeds, raw | Bean, soya, dried |
| Beef, ground, 85% LN meat / 15% fat, raw | Beef, mince, <5% fat, raw |
| Chicken, broilers or fryers, emat and skin, raw | Chicken, flesh, raw |
| Pork, fresh, comp (leg, loin, shoulder, spareribs), lean and fat, raw | Pork, forequarter chop, fully-trimmed, raw |
| Raw whole egg | Egg, chicken, whole, raw |
| Dry heat cooked sockeye salmon | Salmon, baked, roasted, fried, grilled or BBQ'd, no added fat |
| Dry heat cooked atlantic cod | Cod or hake, baked, roasted, fried, grilled or BBQ'd, no added fat |
| Oil, soybean, salad or cooking | Oil, soybean |
| Vegetable oil, canola | Oil, canola |
| Oil, olive, salad or cooking | Oil, olive |
| Oil, vegetable, sunflower, linoleic, (approx 65%) | Oil, sunflower |
| Oil, peanut, salad or cooking | Oil, peanut |
| Salted butter | Butter, plain, salted |
| Oil, vegetable, palm | Oil, palm |
| Lard | Lard |
| Granulated sugar | Sugar, white, granulated or lump |

Supplementary Table 2: Daily average food group intake (servings per day) of Australian adults aged 19-50 years stratified by gender, levels of meat and dairy intake, and indicators of a healthy diet (highest vegetable, lowest discretionary food and beverage consumption, and highest overall diet quality) and compared to the Australian Dietary Guidelines and the Planetary Health Reference Diet. Values are presented as means.

|  | Total | Males | Female | Lowest meat | Highest meat | Lowest dairy | Highest dairy | Lowest vegetables | Highest vegetables | Lowest discretionary | Highest discretionary | Lowest quality | Highest quality | Dietary Guidelines | Planetary Health |
| --- | --- | --- | --- | --- | --- | --- | --- | --- | --- | --- | --- | --- | --- | --- | --- |
| VEGETABLES | 2.72 | 2.82 | 2.62 | 2.22 | 3.47 | 2.70 | 2.90 | 0.81 | 5.71 | 3.22 | 2.23 | 1.64 | 3.92 | 5.50 | 3.83 |
| Starchy veg | 0.55 | 0.57 | 0.53 | 0.34 | 0.77 | 0.60 | 0.58 | 0.04 | 1.39 | 0.61 | 0.49 | 0.29 | 0.83 | 1.13 | 0.44 |
| Other veg | 2.18 | 2.25 | 2.09 | 1.88 | 2.70 | 2.10 | 2.32 | 0.77 | 4.33 | 2.61 | 1.74 | 1.36 | 3.09 | 4.37 | 3.39 |
| FRUIT | 1.44 | 1.49 | 1.39 | 1.60 | 1.40 | 1.34 | 1.57 | 1.41 | 1.64 | 1.60 | 1.26 | 0.91 | 2.12 | 2.00 | 1.33 |
| DAIRY& ALT | 1.55 | 1.71 | 1.39 | 1.55 | 1.53 | 0.53 | 3.21 | 1.49 | 1.70 | 1.55 | 1.50 | 1.42 | 1.71 | 2.50 | 0.96 |
| CEREALS | 4.87 | 5.64 | 4.07 | 4.86 | 4.88 | 4.46 | 5.73 | 4.77 | 5.27 | 5.38 | 4.23 | 4.24 | 5.63 | 6.00 | 7.63 |
| Wholegrains | 1.41 | 1.58 | 1.24 | 1.44 | 1.34 | 1.07 | 1.92 | 1.24 | 1.58 | 1.66 | 1.17 | 0.81 | 2.08 | 3.87 | 3.76 |
| Refined grains | 3.46 | 4.06 | 2.83 | 3.41 | 3.53 | 3.39 | 3.80 | 3.53 | 3.69 | 3.72 | 3.06 | 3.44 | 3.55 | 2.13 | 3.87 |
| MEAT & ALT | 2.31 | 2.76 | 1.84 | 1.09 | 4.59 | 2.38 | 2.25 | 2 | 2.91 | 2.48 | 2.11 | 1.64 | 3.05 | 2.75 | 4.05 |
| Red meat | 1.01 | 1.25 | 0.76 | 0.3 | 2.3 | 0.99 | 1.02 | 0.85 | 1.28 | 1.01 | 1.02 | 0.83 | 1.19 | 0.70 | 0.15 |
| Animal-based alternatives | 1.00 | 1.19 | 0.8 | 0.5 | 1.98 | 1.07 | 0.9 | 0.93 | 1.11 | 1.08 | 0.87 | 0.74 | 1.29 | 0.70 | 0.63 |
| Poultry | 0.68 | 0.82 | 0.54 | 0.3 | 1.43 | 0.75 | 0.64 | 0.65 | 0.73 | 0.73 | 0.64 | 0.57 | 0.85 | 0.23 | 0.25 |
| Fish seafood | 0.19 | 0.22 | 0.17 | 0.12 | 0.34 | 0.19 | 0.16 | 0.18 | 0.25 | 0.24 | 0.11 | 0.09 | 0.28 | 0.23 | 0.28 |
| Eggs | 0.12 | 0.15 | 0.09 | 0.08 | 0.21 | 0.13 | 0.1 | 0.1 | 0.13 | 0.11 | 0.12 | 0.08 | 0.16 | 0.23 | 0.10 |
| Plant-based alternatives | 0.30 | 0.32 | 0.28 | 0.3 | 0.32 | 0.32 | 0.33 | 0.22 | 0.52 | 0.39 | 0.22 | 0.08 | 0.57 | 1.35 | 3.26 |
| Legumes | 0.08 | 0.09 | 0.08 | 0.09 | 0.05 | 0.1 | 0.08 | 0 | 0.22 | 0.1 | 0.06 | 0.03 | 0.12 | 0.63 | 1.59 |
| Nuts | 0.22 | 0.23 | 0.21 | 0.21 | 0.27 | 0.22 | 0.25 | 0.21 | 0.3 | 0.28 | 0.16 | 0.05 | 0.44 | 0.72 | 1.67 |
| UNSATURATED FATS | 2.24 | 2.45 | 2.01 | 1.86 | 2.94 | 2.4 | 2.34 | 1.68 | 3.34 | 2.63 | 1.8 | 1.35 | 3.36 | 4.00 | 5.71 |
| DISCRETIONARY CHOICES | 5.57 | 6.64 | 4.44 | 5.68 | 5.27 | 5.71 | 5.33 | 6.02 | 4.97 | 1.54 | 10.63 | 8.13 | 2.68 | 2.75 | 1.49 |
